# *trans*-Dichlorido(2,2-dimethyl­propane-1,3-diamine)­bis­(triphenyl­phosphane)ruthenium(II)

**DOI:** 10.1107/S1600536810019276

**Published:** 2010-06-05

**Authors:** Monther A. Khanfar, Ismail Warad, Murad A. AlDamen

**Affiliations:** a Department of Chemistry, The University of Jordan, Amman 11942, Jordan; b Department of Chemistry, King Saud University, PO Box 2455, Riyadh-11451, Saudi Arabia

## Abstract

In the title compound, [RuCl_2_(C_5_H_14_N_2_)(C_18_H_15_P)_2_], the Ru^II^ atom is six-coordinated, forming a slightly distorted octa­hedral geometry, with two chloride ions in an axial arrangement, and two P atoms of two triphenyl­phosphane and two chelating N atoms of the bidentate 2,2-dimethyl­propane-1,3-diamine ligand located in the equatorial plane. The average Ru—P, Ru—N and Ru—Cl bond lengths are 2.325 (18), 2.1845 (7) and 2.4123 (12) Å, respectively.

## Related literature

For the reduction of ketones to secondary alcohols, see: Noyori (1994 ▶). For enanti­oselective hydrogenation of prochiral carbonyl compounds to chiral alcohols, see: Drozdzak *et al.* (2005 ▶). For background to stereo-, regio- and enantio-selective ruthenium catalysis, see: Clarke (2002 ▶); Noyori (2003 ▶) and references therein. For Ru^II^ catalysts, see: Noyori & Ohkuma (2001 ▶); Ohkuma *et al.* (2002 ▶); Lindner *et al.* (2005 ▶). For related structures, see: Nachtigall *et al.* (2002 ▶); Lindner *et al.* (2003*a* ▶,*b* ▶); Doucet *et al.* (1998 ▶); Warad *et al.* (2006 ▶).
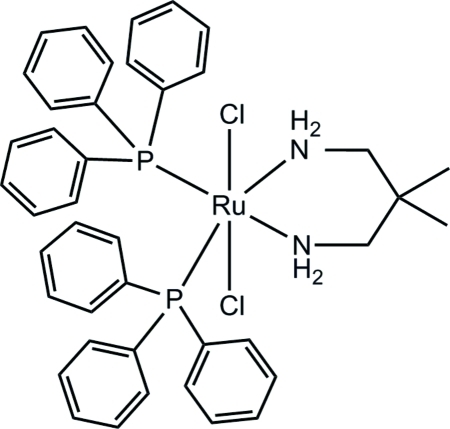


## Experimental

### 

#### Crystal data

[RuCl_2_(C_5_H_14_N_2_)(C_18_H_15_P)_2_]
*M* *_r_* = 798.69
Monoclinic, 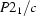

*a* = 17.393 (2) Å
*b* = 10.3493 (16) Å
*c* = 21.315 (2) Å
β = 102.181 (15)°
*V* = 3750.4 (9) Å^3^
*Z* = 4
Mo *K*α radiation
μ = 0.68 mm^−1^
*T* = 293 K
0.60 × 0.60 × 0.05 mm

#### Data collection

Enraf–Nonius CAD-4 diffractometer
Absorption correction: ψ scan (North *et al.*, 1968 ▶) *T*_min_ = 0.687, *T*_max_ = 0.967
7922 measured reflections
7319 independent reflections
5387 reflections with *I* > 2σ(*I*)
*R*_int_ = 0.025
3 standard reflections every 400 reflections  intensity decay: 2%

#### Refinement

*R*[*F* ^2^ > 2σ(*F* ^2^)] = 0.034
*wR*(*F* ^2^) = 0.078
*S* = 1.03
7319 reflections
436 parameters
Only H-atom coordinates refined
Δρ_max_ = 0.39 e Å^−3^
Δρ_min_ = −0.51 e Å^−3^

### 

Data collection: *CAD-4 EXPRESS* (Enraf–Nonius, 1994 ▶); cell refinement: *CAD-4 EXPRESS*; data reduction: *HELENA* (Spek, 1996 ▶); program(s) used to solve structure: *SHELXS97* (Sheldrick, 2008 ▶); program(s) used to refine structure: *SHELXL97* (Sheldrick, 2008 ▶); molecular graphics: *PLATON* (Spek, 2009 ▶); software used to prepare material for publication: *XCIF* in *SHELXTL* (Sheldrick, 2008 ▶).

## Supplementary Material

Crystal structure: contains datablocks global, I. DOI: 10.1107/S1600536810019276/tk2679sup1.cif

Structure factors: contains datablocks I. DOI: 10.1107/S1600536810019276/tk2679Isup2.hkl

Additional supplementary materials:  crystallographic information; 3D view; checkCIF report

## Figures and Tables

**Fig. 1.**
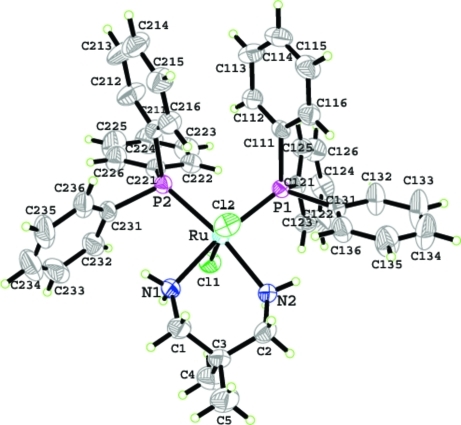
: The structure of the compound, showing 30% probability displacement ellipsoids and the atom numbering scheme.

## supplementary crystallographic information

### Comment

One of the important transformations in the organic synthesis is the reduction of ketones to secondary alcohol (Noyori, 1994). The enantioselective hydrogenation of prochiral carbonyl compounds to chiral alcohols is among the most valuable method in organic chemistry (Drozdzak *et al.*, 2005; Lindner *et al.*, 2005). Furthermore, stereo-, regio- and enantioselective ruthenium-catalysis lies at the heart of current developments in pharmaceutical, agrochemical and similar industries (Noyori, 2003; Clarke, 2002). Recently Noyori *et al.* (Ohkuma *et al.*, 2002; Noyori & Ohkuma, 2001) discovered a ruthenium(II) complex system containing diphosphine and 1,2-diamine ligands which, in the presence of a base and 2-propanol, proved to be excellent catalysts (regarding efficiency, enantioselectivity, and flexibility) for the hydrogenation of ketones under mild conditions (Lindner *et al.*, 2005; Noyori & Ohkuma, 2001). The title complex is crystallized as free solvated *trans*-dichloro-*cis*-bis(triphenylphosphane) isomer with approximate C_2v_ symmetry. The ruthenium atom is coordinated with two chlorine species in *trans* form, one diamine co-ligand *via* the nitrogen atoms and two triphenylphosphane ligands *via* the phosphorus atoms in *cis* forms. The complex exhibits distorted octahedron geometry around the ruthenium center atom with two Ru–N distances of 2.183 (3)Å and 2.185 (3) Å, two Ru–Cl distances of 2.4114 (8)Å and 2.4130 (8)Å and two Ru–P distances equal 2.3120 (8)Å and 2.3370 (8) Å. The diamine and phosphine ligands are practically planar. The coordination angle of the diamine chelate ring results in distinctly N–Ru–N angle of 82.35 (11)° departs from ideal value by up to approximately 7.6°, due to the six-membered ring chelating nature of 1,3-propanediamine ligand, while the P–Ru–P angle is equal 98.55 (3)°. The dichloro ligands are bent away from their axial positions toward the diamine ligand forming Cl–Ru–Cl angle of 166.32 (3)°, resonating to the steric effect of the phenyls in the phosphine ligands. In the crystal structure there are a number of RuCl···HN contacts smaller than 3.0 Å, indicating the presence of unconventional intra-hydrogen bonds (Doucet *et al.*, 1998; Warad *et al.*, 2006).

### Experimental

All the reactions were performed using Schlenk-type flask under argon and standard high vacuum-line techniques. Solvents were of analytical grade and distilled under argon. The title compound was prepared starting from *trans*-RuCl_2_(PPh_3_)_3_ in a similar procedure described previously (Lindner *et al.*, 2003*b*). Mixing of 2,2-dimethylpropane-1,3-diamine (0.059 ml, 0.49 mmol) in dichloromethane (10 ml) dropwise with *trans*-RuCl_2_(PPh_3_)_3_ (0.454 mmol) dissolved in the same solvent (15 ml). The reaction mixture was stirred at room temperature for 2 h. The solvent was removed *in vacuo*. Then the residue was washed well with hexane then diethylether and dried, to yield 310 mg (90%) of yellow powder. The recrystallization was performed by slow diffusion of diethylether into a solution of the complex in dichloromethane to yield orange-brown-plated crystals. 1H NMR (CD_2_Cl_2_): δ (p.p.m.) 0.79 (s, 6H, C(CH_3_)_2_), 2.57 (m, 4H, NCH_2_), 3.12 (br, s, 4H, NH_2_), 7.2–7.7 (m, 20H, C_6_H_5_). ^31^P{1H} NMR (CD_2_Cl_2_):δ (p.p.m.) 46.02 (*s*). _13_C{^1^H} NMR (CD_2_Cl_2_): δ (p.p.m.) 25.3 (s, C(CH_3_)_2_), 34.2 (s, C(CH_3_)_2_), 49.2 (s, CH_2_N), 127.7 (t, N = 4.04 Hz, *m*-C_6_H_5_), 129.3 (s, *p*-C_6_H_5_), 135.4 (t, N=7.42 Hz,*o*-C_6_H_5_), 135.8 (d, N = 18.8 Hz, 1-C_6_H_5_). FAB MS: (m/*z*) 798.1 (*M*^+^). Anal. Calc. for C_41_H_44_Cl_2_N_2_P_2_Ru: C, 66.65.12; H, 5.55; Cl, 8.88; N, 3.51. Found: C, 66.94; H, 5.52; Cl, 9.20; N, 3.59%.

### Refinement

Refinement of F^2^ against ALL reflections. The weighted *R*-factor *wR* and goodness of fit S are based on F^2^, conventional *R*-factors *R* are based on F, with F set to zero for negative F^2^. The threshold expression of F^2^ > 2sigma(F^2^) is used only for calculating *R*-factors(gt) *etc*. and is not relevant to the choice of reflections for refinement. *R*-factors based on F^2^ are statistically about twice as large as those based on F, and R– factors based on ALL data will be even larger. All Hydrogen atoms were refined isotropically.All H atoms were fixed and subsequently refined using a riding model with *U*_iso_(H) = 1.2*U*_eq_ of the carrier atom.

### Crystal data

**Table:**

[RuCl_2_(C_5_H_14_N_2_)(C_18_H_15_P)_2_]	*F*(000) = 1648
*M**_r_* = 798.69	*D*_x_ = 1.411 Mg m^−^^3^
Monoclinic, *P*2_1_/*c*	Mo *K*α radiation, λ = 0.70930 Å
Hall symbol: -P 2ybc	Cell parameters from 25 reflections
*a* = 17.393 (2) Å	θ = 7.8–12.3°
*b* = 10.3493 (16) Å	µ = 0.68 mm^−^^1^
*c* = 21.315 (2) Å	*T* = 293 K
β = 102.181 (15)°	Plate, brown
*V* = 3750.4 (9) Å^3^	0.60 × 0.60 × 0.05 mm
*Z* = 4	

### Data collection

**Table:**

Enraf–Nonius CAD-4 diffractometer	5387 reflections with *I* > 2σ(*I*)
Radiation source: fine-focus sealed tube	*R*_int_ = 0.025
graphite	θ_max_ = 25.9°, θ_min_ = 3.1°
ω scans	*h* = −21→21
Absorption correction: ψ scan (North *et al.*, 1968)	*k* = 0→12
*T*_min_ = 0.687, *T*_max_ = 0.967	*l* = −1→26
7922 measured reflections	3 standard reflections every 400 reflections
7319 independent reflections	intensity decay: 2%

### Refinement

**Table:**

Refinement on *F*^2^	Primary atom site location: structure-invariant direct methods
Least-squares matrix: full	Secondary atom site location: difference Fourier map
*R*[*F*^2^ > 2σ(*F*^2^)] = 0.034	Hydrogen site location: inferred from neighbouring sites
*wR*(*F*^2^) = 0.078	Only H-atom coordinates refined
*S* = 1.03	*w* = 1/[σ^2^(*F*_o_^2^) + (0.027*P*)^2^ + 1.9538*P*] where *P* = (*F*_o_^2^ + 2*F*_c_^2^)/3
7319 reflections	(Δ/σ)_max_ = 0.001
436 parameters	Δρ_max_ = 0.39 e Å^−^^3^
0 restraints	Δρ_min_ = −0.51 e Å^−^^3^

### Special details

**Table:**

Geometry. All e.s.d.'s (except the e.s.d. in the dihedral angle between two l.s. planes) are estimated using the full covariance matrix. The cell e.s.d.'s are taken into account individually in the estimation of e.s.d.'s in distances, angles and torsion angles; correlations between e.s.d.'s in cell parameters are only used when they are defined by crystal symmetry. An approximate (isotropic) treatment of cell e.s.d.'s is used for estimating e.s.d.'s involving l.s. planes.
Refinement. Refinement of *F*^2^ against ALL reflections. The weighted *R*-factor *wR* and goodness of fit *S* are based on *F*^2^, conventional *R*-factors *R* are based on *F*, with *F* set to zero for negative *F*^2^. The threshold expression of *F*^2^ > σ(*F*^2^) is used only for calculating *R*-factors(gt) *etc*. and is not relevant to the choice of reflections for refinement. *R*-factors based on *F*^2^ are statistically about twice as large as those based on *F*, and *R*- factors based on ALL data will be even larger.

### Fractional atomic coordinates and isotropic or equivalent isotropic displacement parameters (Å^2^)

**Table:**

	*x*	*y*	*z*	*U*_iso_*/*U*_eq_	
Ru1	0.293247 (13)	0.49329 (2)	0.344643 (10)	0.02586 (7)	
Cl1	0.39278 (5)	0.65793 (8)	0.37293 (4)	0.0421 (2)	
Cl2	0.22021 (5)	0.29446 (7)	0.32284 (4)	0.0424 (2)	
P1	0.19157 (4)	0.59121 (7)	0.38125 (4)	0.02806 (17)	
C111	0.09258 (16)	0.5977 (3)	0.32882 (14)	0.0316 (7)	
C112	0.07724 (19)	0.6794 (3)	0.27611 (15)	0.0386 (7)	
H11A	0.1169	0.7326	0.2675	0.046*	
C113	0.0032 (2)	0.6825 (3)	0.23610 (17)	0.0494 (9)	
H11B	−0.0067	0.7391	0.2014	0.059*	
C114	−0.0553 (2)	0.6028 (4)	0.24725 (18)	0.0520 (10)	
H11C	−0.1048	0.6050	0.2202	0.062*	
C115	−0.04061 (19)	0.5199 (4)	0.29828 (18)	0.0508 (9)	
H11D	−0.0801	0.4647	0.3054	0.061*	
C116	0.03250 (18)	0.5174 (3)	0.33944 (16)	0.0417 (8)	
H11E	0.0415	0.4616	0.3744	0.050*	
C121	0.20843 (17)	0.7563 (3)	0.41445 (14)	0.0315 (7)	
C122	0.2748 (2)	0.7740 (3)	0.46276 (16)	0.0452 (8)	
H12A	0.3091	0.7052	0.4751	0.054*	
C123	0.2907 (2)	0.8923 (4)	0.49275 (19)	0.0559 (10)	
H12B	0.3345	0.9016	0.5261	0.067*	
C124	0.2429 (2)	0.9950 (4)	0.47395 (19)	0.0562 (10)	
H12C	0.2542	1.0745	0.4942	0.067*	
C125	0.1782 (2)	0.9815 (3)	0.42524 (19)	0.0547 (10)	
H12D	0.1461	1.0523	0.4116	0.066*	
C126	0.1605 (2)	0.8617 (3)	0.39623 (16)	0.0430 (8)	
H12E	0.1156	0.8526	0.3640	0.052*	
C131	0.16632 (17)	0.5131 (3)	0.45301 (14)	0.0350 (7)	
C132	0.1386 (2)	0.5845 (4)	0.49815 (18)	0.0578 (10)	
H13A	0.1363	0.6740	0.4942	0.069*	
C133	0.1142 (3)	0.5270 (4)	0.5490 (2)	0.0828 (16)	
H13B	0.0953	0.5775	0.5785	0.099*	
C134	0.1179 (3)	0.3955 (4)	0.5561 (2)	0.0754 (14)	
H13C	0.1028	0.3568	0.5909	0.090*	
C135	0.1439 (2)	0.3217 (4)	0.5118 (2)	0.0647 (11)	
H13D	0.1453	0.2322	0.5159	0.078*	
C136	0.1682 (2)	0.3799 (3)	0.46079 (17)	0.0485 (9)	
H13E	0.1861	0.3286	0.4311	0.058*	
P2	0.26561 (4)	0.57378 (7)	0.23985 (4)	0.02910 (17)	
C211	0.17237 (18)	0.5472 (3)	0.18165 (14)	0.0337 (7)	
C212	0.1466 (2)	0.6279 (4)	0.12963 (17)	0.0577 (10)	
H21A	0.1754	0.7014	0.1246	0.069*	
C213	0.0781 (3)	0.6003 (5)	0.08490 (18)	0.0713 (13)	
H21B	0.0607	0.6563	0.0507	0.086*	
C214	0.0359 (2)	0.4905 (4)	0.09095 (17)	0.0631 (11)	
H21C	−0.0092	0.4708	0.0603	0.076*	
C215	0.0604 (2)	0.4106 (4)	0.14206 (17)	0.0536 (10)	
H21D	0.0321	0.3360	0.1463	0.064*	
C216	0.1271 (2)	0.4399 (3)	0.18753 (16)	0.0449 (8)	
H21E	0.1419	0.3862	0.2230	0.054*	
C221	0.28413 (18)	0.7468 (3)	0.22798 (15)	0.0344 (7)	
C222	0.26872 (18)	0.8358 (3)	0.27232 (16)	0.0405 (8)	
H22A	0.2537	0.8066	0.3092	0.049*	
C223	0.2753 (2)	0.9669 (3)	0.2627 (2)	0.0540 (10)	
H22B	0.2638	1.0251	0.2927	0.065*	
C224	0.2986 (3)	1.0114 (4)	0.2091 (2)	0.0687 (12)	
H22C	0.3029	1.0997	0.2025	0.082*	
C225	0.3156 (3)	0.9248 (4)	0.1651 (2)	0.0714 (13)	
H22D	0.3320	0.9549	0.1290	0.086*	
C226	0.3088 (2)	0.7939 (3)	0.17389 (18)	0.0527 (9)	
H22E	0.3206	0.7364	0.1437	0.063*	
C231	0.33381 (18)	0.4979 (3)	0.19493 (14)	0.0372 (7)	
C232	0.4127 (2)	0.5332 (4)	0.20829 (18)	0.0529 (10)	
H23A	0.4294	0.6053	0.2336	0.063*	
C233	0.4668 (2)	0.4602 (5)	0.1836 (2)	0.0725 (14)	
H23B	0.5197	0.4830	0.1930	0.087*	
C234	0.4424 (3)	0.3548 (5)	0.1456 (2)	0.0802 (15)	
H23C	0.4788	0.3063	0.1293	0.096*	
C235	0.3650 (3)	0.3213 (4)	0.1316 (2)	0.0735 (13)	
H23D	0.3486	0.2506	0.1052	0.088*	
C236	0.3109 (2)	0.3911 (3)	0.15613 (16)	0.0495 (9)	
H23E	0.2583	0.3665	0.1466	0.059*	
N1	0.38992 (15)	0.3781 (3)	0.32350 (12)	0.0411 (7)	
H1N1	0.3780	0.3623	0.2810	0.049*	
H2N1	0.4325	0.4298	0.3306	0.049*	
N2	0.34248 (15)	0.4290 (3)	0.44251 (12)	0.0383 (6)	
H1N2	0.3807	0.4856	0.4590	0.046*	
H2N2	0.3043	0.4393	0.4647	0.046*	
C1	0.4154 (2)	0.2550 (3)	0.35469 (16)	0.0489 (9)	
H1B	0.4582	0.2214	0.3369	0.059*	
H1C	0.3722	0.1941	0.3445	0.059*	
C2	0.3752 (2)	0.3010 (4)	0.45837 (17)	0.0549 (10)	
H2B	0.3332	0.2382	0.4463	0.066*	
H2C	0.3934	0.2959	0.5046	0.066*	
C3	0.4423 (2)	0.2623 (3)	0.42720 (15)	0.0434 (8)	
C4	0.5121 (2)	0.3503 (4)	0.44572 (19)	0.0664 (12)	
H4A	0.4975	0.4364	0.4311	0.100*	
H4B	0.5539	0.3203	0.4263	0.100*	
H4C	0.5296	0.3505	0.4916	0.100*	
C5	0.4653 (3)	0.1253 (4)	0.4505 (2)	0.0764 (14)	
H5A	0.4828	0.1255	0.4963	0.115*	
H5B	0.5070	0.0950	0.4311	0.115*	
H5C	0.4206	0.0693	0.4386	0.115*	

### Atomic displacement parameters (Å^2^)

**Table:**

	*U*^11^	*U*^22^	*U*^33^	*U*^12^	*U*^13^	*U*^23^
Ru1	0.02516 (11)	0.02601 (12)	0.02646 (11)	0.00136 (11)	0.00557 (8)	0.00011 (11)
Cl1	0.0348 (4)	0.0439 (5)	0.0458 (5)	−0.0102 (4)	0.0044 (3)	0.0005 (4)
Cl2	0.0489 (5)	0.0297 (4)	0.0447 (5)	−0.0058 (4)	0.0014 (4)	−0.0002 (3)
P1	0.0275 (4)	0.0273 (4)	0.0300 (4)	0.0013 (3)	0.0073 (3)	−0.0002 (3)
C111	0.0276 (15)	0.0320 (16)	0.0358 (16)	0.0038 (13)	0.0082 (13)	−0.0051 (13)
C112	0.0390 (18)	0.0373 (18)	0.0391 (18)	0.0005 (15)	0.0074 (14)	−0.0027 (15)
C113	0.051 (2)	0.045 (2)	0.046 (2)	0.0098 (18)	−0.0050 (17)	0.0030 (17)
C114	0.0343 (18)	0.058 (2)	0.058 (2)	0.0082 (18)	−0.0039 (17)	−0.014 (2)
C115	0.0355 (17)	0.058 (2)	0.058 (2)	−0.0098 (17)	0.0094 (16)	−0.007 (2)
C116	0.0353 (16)	0.046 (2)	0.0440 (18)	−0.0061 (16)	0.0079 (14)	−0.0025 (16)
C121	0.0353 (16)	0.0287 (15)	0.0336 (17)	0.0016 (13)	0.0144 (13)	−0.0014 (13)
C122	0.0430 (19)	0.0414 (19)	0.049 (2)	0.0038 (16)	0.0040 (16)	−0.0042 (16)
C123	0.050 (2)	0.055 (2)	0.059 (2)	−0.0112 (19)	0.0025 (18)	−0.018 (2)
C124	0.065 (2)	0.0357 (19)	0.071 (3)	−0.011 (2)	0.021 (2)	−0.022 (2)
C125	0.069 (2)	0.0305 (19)	0.066 (2)	0.0093 (18)	0.017 (2)	−0.0052 (18)
C126	0.0421 (19)	0.0397 (19)	0.046 (2)	0.0055 (15)	0.0079 (16)	−0.0052 (16)
C131	0.0325 (15)	0.0392 (18)	0.0349 (15)	−0.0011 (15)	0.0105 (12)	0.0021 (15)
C132	0.087 (3)	0.041 (2)	0.059 (2)	−0.005 (2)	0.044 (2)	−0.0039 (18)
C133	0.137 (4)	0.057 (3)	0.077 (3)	−0.013 (3)	0.076 (3)	−0.008 (2)
C134	0.108 (4)	0.068 (3)	0.064 (3)	−0.005 (3)	0.051 (3)	0.012 (2)
C135	0.081 (3)	0.047 (2)	0.077 (3)	0.006 (2)	0.041 (2)	0.017 (2)
C136	0.060 (2)	0.0385 (19)	0.055 (2)	0.0082 (17)	0.0294 (19)	0.0069 (17)
P2	0.0320 (4)	0.0273 (4)	0.0286 (4)	0.0006 (3)	0.0079 (3)	0.0012 (3)
C211	0.0368 (17)	0.0363 (16)	0.0278 (15)	0.0032 (14)	0.0064 (13)	−0.0006 (13)
C212	0.058 (2)	0.060 (2)	0.047 (2)	−0.013 (2)	−0.0066 (18)	0.0191 (19)
C213	0.076 (3)	0.086 (3)	0.041 (2)	−0.011 (3)	−0.013 (2)	0.025 (2)
C214	0.056 (2)	0.087 (3)	0.040 (2)	−0.015 (2)	−0.0045 (17)	0.001 (2)
C215	0.049 (2)	0.059 (2)	0.050 (2)	−0.0125 (19)	0.0036 (18)	−0.0034 (19)
C216	0.045 (2)	0.048 (2)	0.0402 (19)	−0.0032 (17)	0.0053 (16)	0.0029 (16)
C221	0.0362 (17)	0.0280 (16)	0.0389 (18)	−0.0026 (13)	0.0075 (14)	0.0050 (14)
C222	0.0386 (18)	0.0346 (17)	0.047 (2)	−0.0010 (15)	0.0074 (15)	0.0031 (15)
C223	0.061 (2)	0.0294 (19)	0.071 (3)	−0.0024 (16)	0.012 (2)	−0.0012 (17)
C224	0.088 (3)	0.031 (2)	0.087 (3)	−0.007 (2)	0.020 (3)	0.017 (2)
C225	0.099 (3)	0.049 (2)	0.074 (3)	−0.006 (2)	0.035 (3)	0.023 (2)
C226	0.071 (3)	0.041 (2)	0.050 (2)	0.0000 (19)	0.0215 (19)	0.0081 (17)
C231	0.0444 (17)	0.0372 (16)	0.0345 (16)	0.0093 (17)	0.0184 (13)	0.0109 (16)
C232	0.052 (2)	0.054 (2)	0.058 (2)	0.0082 (18)	0.0243 (18)	0.0162 (18)
C233	0.051 (2)	0.089 (4)	0.089 (3)	0.017 (2)	0.040 (2)	0.039 (3)
C234	0.099 (4)	0.068 (3)	0.092 (4)	0.039 (3)	0.063 (3)	0.022 (3)
C235	0.108 (4)	0.061 (3)	0.064 (3)	0.023 (3)	0.047 (3)	−0.002 (2)
C236	0.068 (2)	0.044 (2)	0.042 (2)	0.0088 (19)	0.0224 (18)	0.0015 (16)
N1	0.0444 (16)	0.0454 (16)	0.0353 (15)	0.0172 (13)	0.0121 (12)	0.0037 (13)
N2	0.0378 (15)	0.0464 (16)	0.0313 (14)	0.0102 (13)	0.0090 (11)	0.0033 (12)
C1	0.058 (2)	0.043 (2)	0.045 (2)	0.0169 (18)	0.0098 (17)	0.0024 (17)
C2	0.071 (3)	0.056 (2)	0.040 (2)	0.023 (2)	0.0158 (18)	0.0165 (18)
C3	0.0454 (19)	0.046 (2)	0.0381 (18)	0.0177 (16)	0.0075 (15)	0.0056 (16)
C4	0.046 (2)	0.087 (3)	0.061 (3)	0.007 (2)	0.0016 (19)	−0.003 (2)
C5	0.102 (4)	0.064 (3)	0.066 (3)	0.039 (3)	0.022 (3)	0.020 (2)

### Geometric parameters (Å, °)

**Table:**

Ru1—N1	2.184 (2)	C213—C214	1.374 (6)
Ru1—N2	2.185 (2)	C213—H21B	0.9300
Ru1—P1	2.3120 (8)	C214—C215	1.362 (5)
Ru1—P2	2.3370 (8)	C214—H21C	0.9300
Ru1—Cl2	2.4114 (8)	C215—C216	1.379 (5)
Ru1—Cl1	2.4131 (8)	C215—H21D	0.9300
P1—C111	1.845 (3)	C216—H21E	0.9300
P1—C121	1.849 (3)	C221—C222	1.385 (4)
P1—C131	1.863 (3)	C221—C226	1.400 (4)
C111—C112	1.387 (4)	C222—C223	1.381 (4)
C111—C116	1.390 (4)	C222—H22A	0.9300
C112—C113	1.386 (4)	C223—C224	1.371 (5)
C112—H11A	0.9300	C223—H22B	0.9300
C113—C114	1.370 (5)	C224—C225	1.374 (6)
C113—H11B	0.9300	C224—H22C	0.9300
C114—C115	1.366 (5)	C225—C226	1.376 (5)
C114—H11C	0.9300	C225—H22D	0.9300
C115—C116	1.384 (4)	C226—H22E	0.9300
C115—H11D	0.9300	C231—C236	1.387 (5)
C116—H11E	0.9300	C231—C232	1.391 (5)
C121—C126	1.377 (4)	C232—C233	1.393 (5)
C121—C122	1.388 (4)	C232—H23A	0.9300
C122—C123	1.382 (5)	C233—C234	1.371 (7)
C122—H12A	0.9300	C233—H23B	0.9300
C123—C124	1.356 (5)	C234—C235	1.361 (7)
C123—H12B	0.9300	C234—H23C	0.9300
C124—C125	1.368 (5)	C235—C236	1.375 (5)
C124—H12C	0.9300	C235—H23D	0.9300
C125—C126	1.391 (5)	C236—H23E	0.9300
C125—H12D	0.9300	N1—C1	1.461 (4)
C126—H12E	0.9300	N1—H1N1	0.9000
C131—C132	1.378 (4)	N1—H2N1	0.9000
C131—C136	1.387 (4)	N2—C2	1.453 (4)
C132—C133	1.380 (5)	N2—H1N2	0.9000
C132—H13A	0.9300	N2—H2N2	0.9000
C133—C134	1.369 (6)	C1—C3	1.520 (4)
C133—H13B	0.9300	C1—H1B	0.9700
C134—C135	1.363 (5)	C1—H1C	0.9700
C134—H13C	0.9300	C2—C3	1.514 (5)
C135—C136	1.386 (5)	C2—H2B	0.9700
C135—H13D	0.9300	C2—H2C	0.9700
C136—H13E	0.9300	C3—C4	1.502 (5)
P2—C211	1.843 (3)	C3—C5	1.527 (5)
P2—C221	1.846 (3)	C4—H4A	0.9600
P2—C231	1.849 (3)	C4—H4B	0.9600
C211—C216	1.383 (4)	C4—H4C	0.9600
C211—C212	1.385 (4)	C5—H5A	0.9600
C212—C213	1.389 (5)	C5—H5B	0.9600
C212—H21A	0.9300	C5—H5C	0.9600
			
N1—Ru1—N2	82.35 (9)	C212—C213—H21B	119.9
N1—Ru1—P1	170.31 (7)	C215—C214—C213	119.6 (3)
N2—Ru1—P1	89.14 (7)	C215—C214—H21C	120.2
N1—Ru1—P2	90.54 (7)	C213—C214—H21C	120.2
N2—Ru1—P2	168.90 (7)	C214—C215—C216	120.3 (4)
P1—Ru1—P2	98.55 (3)	C214—C215—H21D	119.9
N1—Ru1—Cl2	83.77 (8)	C216—C215—H21D	119.9
N2—Ru1—Cl2	90.48 (8)	C215—C216—C211	121.6 (3)
P1—Ru1—Cl2	91.70 (3)	C215—C216—H21E	119.2
P2—Ru1—Cl2	97.25 (3)	C211—C216—H21E	119.2
N1—Ru1—Cl1	83.92 (8)	C222—C221—C226	117.9 (3)
N2—Ru1—Cl1	81.99 (8)	C222—C221—P2	119.1 (2)
P1—Ru1—Cl1	99.54 (3)	C226—C221—P2	122.9 (3)
P2—Ru1—Cl1	88.81 (3)	C223—C222—C221	121.2 (3)
Cl2—Ru1—Cl1	166.33 (3)	C223—C222—H22A	119.4
C111—P1—C121	104.52 (14)	C221—C222—H22A	119.4
C111—P1—C131	99.29 (13)	C224—C223—C222	120.2 (4)
C121—P1—C131	97.63 (14)	C224—C223—H22B	119.9
C111—P1—Ru1	119.59 (9)	C222—C223—H22B	119.9
C121—P1—Ru1	117.61 (10)	C223—C224—C225	119.6 (4)
C131—P1—Ru1	114.61 (10)	C223—C224—H22C	120.2
C112—C111—C116	118.3 (3)	C225—C224—H22C	120.2
C112—C111—P1	120.5 (2)	C224—C225—C226	120.8 (4)
C116—C111—P1	121.1 (2)	C224—C225—H22D	119.6
C113—C112—C111	120.5 (3)	C226—C225—H22D	119.6
C113—C112—H11A	119.8	C225—C226—C221	120.3 (4)
C111—C112—H11A	119.8	C225—C226—H22E	119.8
C114—C113—C112	120.5 (3)	C221—C226—H22E	119.8
C114—C113—H11B	119.8	C236—C231—C232	118.6 (3)
C112—C113—H11B	119.8	C236—C231—P2	120.8 (3)
C115—C114—C113	119.7 (3)	C232—C231—P2	119.7 (3)
C115—C114—H11C	120.1	C231—C232—C233	119.7 (4)
C113—C114—H11C	120.1	C231—C232—H23A	120.1
C114—C115—C116	120.6 (3)	C233—C232—H23A	120.1
C114—C115—H11D	119.7	C234—C233—C232	120.3 (4)
C116—C115—H11D	119.7	C234—C233—H23B	119.8
C115—C116—C111	120.5 (3)	C232—C233—H23B	119.8
C115—C116—H11E	119.8	C235—C234—C233	120.1 (4)
C111—C116—H11E	119.8	C235—C234—H23C	120.0
C126—C121—C122	117.7 (3)	C233—C234—H23C	120.0
C126—C121—P1	125.9 (2)	C234—C235—C236	120.5 (4)
C122—C121—P1	116.5 (2)	C234—C235—H23D	119.7
C123—C122—C121	120.9 (3)	C236—C235—H23D	119.7
C123—C122—H12A	119.6	C235—C236—C231	120.8 (4)
C121—C122—H12A	119.6	C235—C236—H23E	119.6
C124—C123—C122	120.5 (3)	C231—C236—H23E	119.6
C124—C123—H12B	119.8	C1—N1—Ru1	123.7 (2)
C122—C123—H12B	119.8	C1—N1—H1N1	106.4
C123—C124—C125	120.0 (3)	Ru1—N1—H1N1	106.4
C123—C124—H12C	120.0	C1—N1—H2N1	106.4
C125—C124—H12C	120.0	Ru1—N1—H2N1	106.4
C124—C125—C126	119.8 (3)	H1N1—N1—H2N1	106.5
C124—C125—H12D	120.1	C2—N2—Ru1	123.7 (2)
C126—C125—H12D	120.1	C2—N2—H1N2	106.4
C121—C126—C125	121.1 (3)	Ru1—N2—H1N2	106.4
C121—C126—H12E	119.4	C2—N2—H2N2	106.4
C125—C126—H12E	119.4	Ru1—N2—H2N2	106.4
C132—C131—C136	116.9 (3)	H1N2—N2—H2N2	106.5
C132—C131—P1	121.2 (3)	N1—C1—C3	114.7 (3)
C136—C131—P1	121.7 (2)	N1—C1—H1B	108.6
C131—C132—C133	121.8 (4)	C3—C1—H1B	108.6
C131—C132—H13A	119.1	N1—C1—H1C	108.6
C133—C132—H13A	119.1	C3—C1—H1C	108.6
C134—C133—C132	120.0 (4)	H1B—C1—H1C	107.6
C134—C133—H13B	120.0	N2—C2—C3	116.1 (3)
C132—C133—H13B	120.0	N2—C2—H2B	108.3
C135—C134—C133	119.7 (4)	C3—C2—H2B	108.3
C135—C134—H13C	120.1	N2—C2—H2C	108.3
C133—C134—H13C	120.1	C3—C2—H2C	108.3
C134—C135—C136	120.0 (4)	H2B—C2—H2C	107.4
C134—C135—H13D	120.0	C4—C3—C2	112.3 (3)
C136—C135—H13D	120.0	C4—C3—C1	110.9 (3)
C135—C136—C131	121.5 (3)	C2—C3—C1	111.0 (3)
C135—C136—H13E	119.2	C4—C3—C5	109.6 (3)
C131—C136—H13E	119.2	C2—C3—C5	106.1 (3)
C211—P2—C221	101.92 (14)	C1—C3—C5	106.7 (3)
C211—P2—C231	99.08 (14)	C3—C4—H4A	109.5
C221—P2—C231	101.03 (14)	C3—C4—H4B	109.5
C211—P2—Ru1	124.38 (10)	H4A—C4—H4B	109.5
C221—P2—Ru1	118.04 (10)	C3—C4—H4C	109.5
C231—P2—Ru1	108.57 (10)	H4A—C4—H4C	109.5
C216—C211—C212	117.5 (3)	H4B—C4—H4C	109.5
C216—C211—P2	119.7 (2)	C3—C5—H5A	109.5
C212—C211—P2	122.7 (3)	C3—C5—H5B	109.5
C211—C212—C213	120.8 (4)	H5A—C5—H5B	109.5
C211—C212—H21A	119.6	C3—C5—H5C	109.5
C213—C212—H21A	119.6	H5A—C5—H5C	109.5
C214—C213—C212	120.3 (4)	H5B—C5—H5C	109.5
C214—C213—H21B	119.9		
